# Non-attendance of mammographic screening: the roles of age and municipality in a population-based Swedish sample

**DOI:** 10.1186/s12939-015-0291-7

**Published:** 2015-12-30

**Authors:** Maria Norfjord Zidar, Peter Larm, Per Tillgren, Sharareh Akhavan

**Affiliations:** Division of Public Health Sciences, School of Health, Care and Social Welfare, Mälardalen University, Box 883, SE-721 23 Västerås, Sweden; Centre for Clinical Research, County Council of Västmanland and Uppsala University, Uppsala, Sweden

**Keywords:** Age, Distance, Equitable health care, Mammographic screening, Municipality of residence, Sweden

## Abstract

**Background:**

Inequality in health and health care is increasing in Sweden. Contributing to widening gaps are various factors that can be assessed by determinants, such as age, educational level, occupation, living area and country of birth. A health care service that can be used as an indicator of health inequality in Sweden is mammographic screening. The non-attendance rate is between 13 and 31 %, while the average is about 20 %. This study aims to shed light on three associations: between municipality and non-attendance, between age and non-attendance, and the interaction of municipality of residence and age in relation to non-attendance.

**Methods:**

The study is based on data from the register that identifies attenders and non-attenders of mammographic screening in a Swedish county, namely the Radiological Information System (RIS). Further, in order to provide a socio-demographic profile of the county’s municipalities, aggregated data for women in the age range 40–74 in 2012 were retrieved from Statistics Sweden (SCB), the Public Health Agency of Sweden, the National Board of Health and Welfare, and the Swedish Social Insurance Agency. The sample consisted of 52,541 women. Analysis conducted of the individual data were multivariate logistic regressions, and pairwise chi-square tests.

**Results:**

The results show that age and municipality of residence associated with non-attendance of mammographic screening. Municipality of residence has a greater impact on non-attendance among women in the age group 70 to 74. For most of the age categories there were differences between the municipalities in regard to non-attendance to mammographic screening.

**Conclusions:**

Age and municipality of residence affect attendance of mammographic screening. Since there is one sole and pre-selected mammographic screening facility in the county, distance to the screening facility may serve as one explanation to non-attendance which is a determinant of inequity. From an equity perspective, lack of equal access to health and health care influences facility utilization.

## Background

Access to health care services concern equal use for equal need. National mammographic screening programmes could be considered equal as it invites all women in a certain age group to participate. Everyone also has the right to decline, however if the decline to attend is due to barriers that can be adjusted then it becomes a matter of inequity in health care [1].

In Sweden, all women between 40 and 74 years receive an invitation to participate in screening every 18 to 24 months, depending on the county they live in [2]. Approximately 20 % of invited women decline the invitation. Since the patient fee for mammographic screening in Sweden is low, ranging from free-of-charge to 200 SEK [3] depending on county council, other individual and structural reasons for declining may be at work and could be a marker of inequity. This is of importance to investigate since breast cancer is the most common type of cancer in women, and was ranked as the fifth cause of death from cancer globally in 2012 [4]. The incidence of breast cancer among women in Sweden in 2011 represented 30.3 % of total cases of cancer [5], and an important service for the early detection of potential breast cancer is mammographic screening. Previous studies have shown a decrease in breast cancer mortality among women participating in screening [6–9], beneficial for public health since it may lead to early treatment and is cost-effective [10]. However, the benefits of mammographic screening have been questioned, mainly because of the risks of over-diagnosis and unnecessary treatment [11]. Previous studies in a variety of countries have investigated attendance of mammographic screening, with a focus on different demographic or socio-economic determinants. These determinants of health can, from an equity perspective, influence health at a social, economic, and individual level [12]. For instance being older has been found to be associated with a higher rate of non-attendance [13–15] as well as being younger [16]. Women who have never been married, or are separated or divorced, have a lower rate of attendance [13, 17]. More education and higher income are associated with a higher probability of attendance [13, 18]. Residential area has also been shown to affect the likelihood of attendance, which can differ both within a city and between rural and urban places of residence [18, 19]. Regional level of income and education may further explain non-attendance of mammographic screening [14], as too may a region’s socio-economic level of deprivation, as measured by proportions of over-crowded households, economically active residents unemployed, and households not in owner-occupancy [20].

In addition, travel time and parking facilities are factors that impact on non-attendance [21]. This may be assumed to coincide with geographic location in the context of distance to a mammographic facility [14, 17, 20, 22, 23], where even living more than 5 km from the screening facility has been found to have a negative impact on attendance [17]. Also, shorter distance to a mammographic facility has been found to have an effect in terms of attracting not previously screened women from socially disadvantaged areas [24]. With regard to ethnicity, a recurring finding is that not being native-born correlates with a lower degree of attendance [13, 17], which is congruent with the findings of a Swedish study [25].

Swedish studies of mammographic screening have focused mainly on its effectiveness in preventing breast-cancer mortality [7, 26–28], and the determinants of non-attendance [25, 29–31]. These studies show that marital status, unemployment, income, housing situation, level of education [25, 30], and having no children or five children or more [30] are associated with non-attendance. Practical reasons affecting attendance of screening are concerned with logistics, such as time from work and travelling time [32], out-of-pocket expenses, and not being able to choose mammographic facility (when one has been pre-selected) [33]. To the best of our knowledge, no study has been conducted in a county in Sweden where all women are invited to the sole and pre-selected mammographic facility. This study aims to shed light on three associations: between municipality and non-attendance, between age and non-attendance, and the interaction of municipality of residence and age in relation to non-attendance.

## Methods

### Design and setting

A study with a cross-sectional design was performed since our data only covered the years 2011/2012 for a Swedish county and its municipalities. The county is divided into 10 municipalities and in 2012, the total population (men and women in all age groups) was 256,224 [34] and the total amount of women invited between the age 40–74 was 52,541. The sole mammographic screening facility in the county is situated in its only urban municipality (defined as a municipality with a population of ≥30,000 and/or where the main densely populated area consists of a population of ≥25,000). It is not optional to choose another counties mammographic facility as no agreements have been made between the studied county and other counties. The remaining nine municipalities are rural, which means that they do not meet the criteria of being a metropolitan or an urban area, but have a population density of at least five inhabitants/km^2^ [35]. The use of distance is to give a description of how each municipality is located in relation to the sole and pre-selected mammographic facility that women are invited to.

### Participants

The present study, conducted in a medium-sized county in Sweden, included all the women from 40 to 74 years of age who were invited to mammographic screening in 2011/2012. The total of invited women were 52,861 and of these women 320 was calculated as missing due to for instance that the municipality of residence was not accounted for. This resulted in a sample of 52,541 women, of whom 42,570 (81 %) attended and 9971 (19 %) were non-attenders. Women in the county are invited biannually to screening (on an alternate-year basis), hence 2 years (2011/2012) account for the total population of invitees. Also included were women over 74 years of age when they responded to an invitation.

### Measures

The independent variables, municipality and age, and the dependent variable, non-attendance of mammographic screening are based on micro-level data. However, in order to describe the municipalities, the socio-demographic characteristics (ten chosen variables) of each municipality are presented on the basis of macro-level data.

#### Non-attendance of mammographic screening

Data were collected from the Radiological Information System (RIS) to identify attenders and non-attenders, between the ages of 40 and <74 years, residing in the county during the years 2011/2012 [36]. The RIS is the administrative register at the local mammographic screening facility, where information about invitations, and participation and declining, is stored. Women who were invited but did not attend screening during the years 2011/2012 were counted as non-attenders.

#### Municipality of residence

Municipality of residence was identified by postal codes extracted from the RIS.

#### Age

The ages of the women were also extracted from the RIS and grouped into the following categories: 40–44, 45–49, 50–54, 55–59, 60–64, 65–69, 70 and above.

#### Socio-demographic characteristics of the municipalities

Data at municipality level were taken from Statistics Sweden, the Public Health Agency of Sweden, the National Board of Health and Welfare, and the Swedish Social Insurance Agency. Nine variables were selected at municipality level to represent socio-demographic characteristics and are displayed in Table 1*. Labour position* can be either gainfully employed or not gainfully employed [37] (in Table 1 only the “gainfully employed” are included). *Level of income of women is* accounted for by presenting “highest share of low income earner”. The limit for being classified as a low income earner is when the person’s total income is less than 20 % of all the income earners in the country. The limit applies to the earned income of age group 20–64 years, and includes all income earners, including those with no income [38]. *Educational level* was established by allocating the initial eight levels in the Swedish National Educational Classification (SUN) to four clusters: A. Low (≤10 years), B. Middle (11–15 years) [39], C: High >15 years), and D. No information about level of educational attainment [40].

**Table 1:** Socio-demographic characteristics of the municipalities, women aged 40 to 74 in 2012

Municipality	A		B		C		D		E		F		G		H		I		J	
Urban/Rural area	Urban		Rural		Rural		Rural		Rural		Rural		Rural		Rural		Rural		Rural	
Distance to the sole mammographic facility	km <19		km 20–39		km 20–39		km 40–59		km 40–59		km 40–59		km 40–59		km 60–79		km 60–79		km 60–79	
Non-attendance (*N*, %)	*N*	%	*N*	%	*N*	%	*N*	%	*N*	%	*N*	%	*N*	%	*N*	%	*N*	%	*N*	%
	4953	18.0	376	17.6	612	18.8	579	19.9	936	19.9	1164	20.9	357	21.5	522	19.6	213	24.4	259	21.7
Labour position (%)																				
Gainfully employed	60.38		54.22		56.11		56.52		61.71		58.88		58.49		57.82		55.66		58.68	
Highest share of low income earner (%) (women, aged 20–64)	21.71		19.38		21.10		21.76		20.00		22.26		18.36		21.48		20.51		20.36	
Educational level (%)																				
A ≤10 years	16.84		24.90		28.11		21.75		16.63		20.51		21.35		27.76		25.58		23.66	
B 11–15 years	60.36		63.52		64.61		62.17		67.24		65.06		63.55		63.56		61.40		66.46	
C >15 years	21.93		10.92		12.50		15.52		15.43		13.67		13.99		11.95		12.47		11.64	
D No information	0.85		0.65		0.94		0.54		0.69		0.74		1.09		0.72		0.53		0.22	
Civil status (%)																				
Unmarried/divorced/ widowed	45.40		40.91		43.21		44.34		46.25		45.16		41.67		47.70		46.08		46.29	
Married	54.59		58.92		56.61		55.72		53.73		54.64		58.07		52.29		54.19		53.71	
Health (days)																				
Incapacity measure - number of days of sick-leave	181.99		233.85		212.39		174.19		190.04		184.43		195.71		198.79		211.98		195.58	
Type of household (%)																				
Renting	21.95		13.59		24.17		29.70		27.61		26.39		22.58		13.95		19.80		19.25	
Ownership/right in a co-operative building society dwelling	71.35		83.17		70.90		65.76		67.87		69.96		74.18		82.27		75.53		77.35	
Special/Other building/ Information missing	6.70		3.24		4.94		4.53		4.51		3.65		3.24		3.78		4.66		3.40	
Ethnicity (%)																				
Foreign-born women in the female population, age group 35–65+	21.42		25.83		21.66		11.88		10.12		20.58		15.51		24.27		20.00		13.46	
Family size (%)																				
0 children	78.16		78.99		79.40		79.85		79.45		80.09		78.83		80.60		83.69		81.87	
1–3 children	21.26		20.23		19.62		19.51		19.94		19.22		20.24		18.73		15.79		17.20	
4 or more children	0.57		0.79		0.98		0.64		0.60		0.69		0.94		0.68		0.52		0.93	
Age (%)																				
40–49	33.07		27.17		29.49		28.63		28.45		29.83		28.25		29.07		23.07		26.34	
50–59	26.99		26.74		27.11		25.71		28.65		28.25		27.26		26.15		29.71		29.67	
60–69	28.95		34.51		30.78		33.03		31.59		30.51		33.00		31.49		34.56		33.46	
70–74	10.99		11.58		12.62		12.63		11.30		11.41		11.49		13.29		12.66		10.53	

Note. Number of invited, attending and non-attending, women, total: 52,541. In some instances, age range differs from 40 to 74 due to the type of variable that has been investigated

Educational level: A: Low (≤10 years), B: Middle (11–15 years), C: High >15 years), and D: No information about level of educational attainment

*Civil status* was merged into two groups: unmarried, divorced or widowed as one group, and married as the other [39]. *Health* was assessed according to the Swedish Social Insurance Agency’s incapacity measures, which account for the number of days of sick-leave covered by the insurance over a 12-month period [41]. A percentage was calculated for each municipality. *Type of household* was first categorized into eight groups and then condensed into three: renting, ownership/right in a co-operative building society (i.e., joint ownership), and special dwellings/other housing/information missing [42]. *Ethnicity* was measured as the percentage of foreign-born women in the female population in the age range 35–65+, since figures for the age range 40 to 74 were not available [43]. *Family size* was categorized by number of children, covering families with no children to families with four or more [38]. *Distance* (km) was estimated using the service provided by Google maps, referencing from the train station in each municipality to the postal address where the sole (hence pre-selected) mammographic facility in the county is located. Distances were grouped into the following four categories: <19, 20–39, 40–59, 60–79 km.

### Procedure

The procedure consisted of two separate processes, one analytic and one descriptive. The analytic part involved individual retrieval of data from the RIS to identify attenders and non-attenders of mammographic screening in the studied county in 2011/2012. In addition, information on the postal code and age of each woman was extracted from the RIS. Then, by using the postal code, each woman was identified as the resident of a particular municipality. The second step was to aggregate macro-level data on socio-demographic factors for each municipality in the studied county for women in the age range 40–74 in 2012 (if nothing else is stated). Ethical approval for the study was obtained from the Swedish Ethical Board–Uppsala (Dnr. 2013/071).

### Statistical analysis

First, the prevalence of non-attendance in each municipality was calculated. Second, the independent associations between municipality and non-attendance and between age and non-attendance were assessed in a multivariate logistic regression analysis. In the logistic regressions non-attenders were coded as “1” and attenders as “0”. Consequently when the logistic regressions is presented in the text only the non-attenders are mentioned. The municipality where the sole mammographic facility is situated was used as the reference area. We needed to examine whether non-attendance of mammographic screening differed in the municipalities of the county as compared with the municipality where the screening facility is situated. Third, in order to examine whether the prevalence of non-attendance in different municipalities differed between age categories (with the youngest age group, 40–44, as the reference age category), pairwise chi-square tests between the nine municipalities and the municipality where the mammographic screening facility is situated (the reference age category) were performed. Fourth, the moderating effect of age on the association between municipalities and non-attendance was examined as an interaction effect using multivariate logistic regression analysis. This analysis concerned whether the associations between each municipality as compared with the reference municipality and non-attendance differed according to age. In the interaction analysis, age was calculated as a continuous rather than a categorical variable in order to increase statistical power. All analyses were performed using the Statistical Package for the Social Sciences, SPSS Statistics, version 19, by IBM.

## Results

### Prevalence of non-attendance and description of the municipalities

The non-attendance rate, municipality of residence classified as urban or rural, distance to the municipality where the mammographic facility is situated, and values on the socio-demographic variables for the different municipalities are shown in Table 1. The range of non-attendance is between 17.6 and 24.4 %, with an average of 20 %.

Municipality A, where the mammographic facility is located hence the reference municipality, is the only urban area in the county and is socio-economically well positioned, with the youngest female population, the highest education level, the second highest employment rate, and the second lowest sick-leave rate. Municipality B, a rural area, has the lowest rate of non-attendance (17.6 %) and is located 20–39 km from the facility. This municipality is characterized by low scores on the socio-economic indexes, including the lowest employment rate, the highest number of days of sick-leave among the female population in 2012, and the lowest number of women with a high education (10.92 %). In addition, this municipality has the highest proportion of foreign-born women in the female population aged 35 or older, and the highest percentage of married couples (58.92 %). By contrast, Municipality I, with the highest rate of non-attending women (24.4 %), and also a rural area, is located 60–79 km from the mammographic facility. This municipality is characterized by low scores on the socio-economic indexes, including the second lowest employment rate, and is in third place regarding education and sick-leave. In addition, the women living in Municipality I have no children to a greater extent than the women living in the other municipalities in the county, and they are also older.

#### Non-attendance in relation to municipality of residence

As compared with Municipality A, women in all the other municipalities in the county display an increased probability of non-attendance, with the exception of women in the two geographically closest municipalities (B and C). Whereas women in all the municipalities situated more than 40 km from the facility showed an increased probability of not attending, the greatest probability was shown by two municipalities situated 60–79 km from Municipality A, with increased odds of 54 % for Municipality I, and 30 % for Municipality J (Table 2).

**Table 2:** The association between municipality of residence and non-attendance of mammographic screening assessed in a multivariate logistic regression analysis adjusting for age (*n* = 52,541)

Municipality	%	Estimated distance (km) to mammographic facility	OR (95 % CI)	*P*
A (ref.)	52.5	<19 (ref.)	1.00 (reference)	
B	4.1	20–39	1.01 (0.90–1.13)	0.998
C	6.2	20–39	1.08 (0.98–1.18)	0.131
D	5.5	40–59	1.17 (1.06–1.29)	0.002
E	9.0	40–59	1.15 (1.07–1.25)	0.001
F	10.6	40–59	1.23 (1.14–1.32)	< 0.001
G	3.2	40–59	1.28 (1.13–1.44)	< 0.001
H	5.1	60–79	1.13 (1.02–1.25)	0.017
I	1.7	60–79	1.54 (1.32–1.81)	< 0.001
J	2.3	60–79	1.30 (1.13–1.50)	< 0.001

Note. Ref. refers to the municipality in which the mammographic facility is located. The percentage is accounting for all the women living in each municipality, in relation to the total sample

#### Non-attendance in relation to age

Generally, the association between age and non-attendance after adjusting for municipality shows that the probability of not attending mammographic screening decreased with age as compared with the reference age group (40–44). The magnitude of the decrease in probability with age appears to be linear but has not been tested, with the exception of the oldest age category (70 years or above), which indicates that women attend mammographic screening to a greater extent as they become older (Table 3).

**Table 3:** Non-attendance in relation to age in the studied county (*n* = 52,541)

Age category	%	Crude OR (95 % CI)	*P*	Adjusted OR (95 % CI)	*P*
40–44 (ref.)	12.8	1.00 (reference)	0.003	1.00 (reference)	0.002
45–49	16.6	0.89 (0.83–0.96)	< 0.001	0.89 (0.82–0.96)	< 0.001
50–54	14.3	0.83 (0.77–0.90)	< 0.001	0.82 (0.76–0.89)	< 0.001
55–59	13.1	0.70 (0.64–0.76)	< 0.001	0.69 (0.64–0.75)	< 0.001
60–64	14.8	0.58 (0.53–0.63)	< 0.001	0.57 (0.53–0.62)	< 0.001
65–69	15.7	0.51 (0.47–0.55)	< 0.001	0.50 (0.46–0.55)	< 0.001
70–	12.8	0.67 (0.62–0.73)	< 0.001	0.67 (0.61–0.72)	< 0.001

Note. Adjusted OR is adjusted for municipalities. The percentage is accounting for all the women in each age category, in relation to the total sample

#### The relation between municipality and age with regard to non-attendance

Non-attendance in the surrounding municipalities as compared to the reference municipality was in general statistically higher in the following age categories: 45–49 for four municipalities, 50–54 for three municipalities, 65–69 for five municipalities, and ≥70 for seven municipalities (Table 4).

**Table 4:** The prevalence of non-attendance in percent, separately for each municipality and age category

Age	A (ref.) km <19	B km 20–39	C km 20–39	D km 40–59	E km 40–59	F km 40–59	G km 40–59	H km 60–79	I km 60–79	J km 60–79
40–44	23.3	22.3	23.4	20.7	25.8	28.7**	25.5	26.0	30.1	26.6
45–49	21.1	17.1*	24.6*	22.5	24.4*	23.3	27.2*	25.5*	27.2	20.7
50–54	20.2	18.5	19.6	22.6	19.6	24.0*	30.5***	16.4*	30.3**	25.5
55–59	17.9	18.2	20.1	22.8*	16.9	18.4	19.7	16.3	23.4	17.7
60–64	14.9	19.9*	15.2	16.7	15.5	16.6	16.6	14.3	19.1	18.5
65–69	12.9	12.4	14.1	16.7*	15.9*	14.9	10.7	17.7**	18.8*	17.4***
70–	14.6	17.6	15.7	19.0*	23.1***	22.3***	22.0**	20.8**	26.2**	29.2**

Note. Difference in prevalence between each municipality and the reference municipality was calculated using a pairwise Chi square test, df = 1, two-tailed

*P ≤ 0.05, **P ≤ 0, 01, ***P ≤ 0.001

The ORs between municipalities and non-attendance differed according to age, and were assessed as interaction effects. Four of the interactions were significant, indicating a stronger association between municipalities B, D, E, and J and non-attendance at older compared with younger ages (Table 5). In other words, by contrast with the general trend of a lower prevalence of non-attendance with increased age, the probability of not attending mammographic screening is higher among older women than younger ones in these municipalities.

**Table 5:** Interaction effects of age on municipality for odds ratios (OR) of non-attendance and 95 % confidence intervals (CI)

Municipality	OR (95 % CI)
A (ref.)	1.00 (reference)
B × Age	1.014 (1.002–1.026)*
C × Age	1.000 (0.991–1.010)
D × Age	1.014 (1.004–1.024)*
E × Age	1.008 (1.000–1.016)*
F × Age	1.005 (0.998–1.013)
G × Age	0.998 (0.986–1.011)
H × Age	1.008 (0.998–1.018)
I × Age	1.009 (0.993–1.026)
J × Age	1.018 (1.003–1.032)*

Note. *statistically significant. Age in these interaction effects are calculated as continuously variables instead of categorical

## Discussion

This study describes and analyses reasons for non-attendance of mammographic screening using age, municipality of residence where distance from the sole and pre-selected facility in the county serves to give an understanding of the context, and aggregated socio-demographic variables as factors of interest in a cross-sectional design. Even though some of the variables are at municipality level, the study may contribute to further understanding of the roles of different socio-demographic factors and their composition on non-attendance of screening. The results show that age and municipality of residence has an impact on attendance of mammographic screening, but municipality have a greater effect on women in the age group 70–74. Distance may have an impact as it could affect the attendance where a longer distance correspond with a lower participation rate. The impact of age [13, 15, 44], and also the effect of distance [13–15], on mammographic screening attendance are supported in several previous studies. The estimated threshold for attendance regarding distance to a mammographic screening facility has previously been found to vary for distances between 3 and 25 km [13, 24, 45], whereas the current study indicates that a distance >40 km negatively affects attendance. The reasons for this threshold distance of >40 km may be found in the socio-demographic profiles of the municipalities, as well as in infrastructural conditions. This could reflect a contextual effect which concern cultural, political, or institutional context [46] and is a social determinant of health referencing the main determinants of health by Dahlgren and Whitehead [47]. Social inequity in health arises when it is systematic, socially produced and unfair [48]. It would be of interest further to investigate deviations from the trend of a negative effect of increased distance on attendance.

Based on previous findings, certain socio-demographic factors can be seen as predictors of the likelihood of attending different health screening services, so looking at the municipalities that deviate may reveal certain factors that encourage or discourage attendance.

In this study, age is indicated as playing a role with regard to attendance, especially among women aged over 65 who reside in a municipality that are located further away from the mammographic screening facility. Travel distance and also the cost involved may be deterrents to attending mammographic screening. This is given support, since there are previous indications that distance to mammographic facility may exacerbate barriers for women living further away from the facility [13, 49].

The age effect on attendance found in this study is in line with the results of previous research in the field, since some studies have found that higher ages, and others younger ages, are favoured in relation to non-attendance of mammography [13–16]. When distance is taken into account, the current study shows that women aged 70 or more living in municipalities further than 40 km from where the mammographic screening facility is located, decline invitations to screening more often than women in younger age groups. Coincidently or not, it is nevertheless of importance to consider. If the reason to not attend is due to geographical location, or social, or economic obstacles, even if there initially was a wish to attend, then it is unfair [1]. Additionally, from the perspective of equity, this is a problem, since a lack of equality of access to health and health care influences the utilization of health care facilities. Rural women, in comparison with urban women, have been found not to attend mammographic screening to a greater extent [19, 50]. The choice to settle in areas that are more rural, less populated, and further away from what can be considered as the central municipality in a county is optional. Guiding this choice are factors like availability of affordable housing. The housing market for the urban area in the county shows a shortage of accommodation [51], which may lead to a flow of people to other municipalities in the county. If the threshold distance to travel to attend mammographic screening is >40 km, the need for preparedness is important. Opening more screening facilities at different locations in the county may be a solution worth considering. This is also suggested in a previous paper as one approach to improve the attendance rate and reduce geographic inequities in mammographic screening [52].

In addition, increase in life expectancy is not taken into account with regard to the upper age limit for attending the organized mammographic screening program. The life expectancy of Swedish women in 2012 was 83.4 years and is predicted to rise to 89 years by 2060 [53]. Remaining years of potential ill-health are extended not only if women aged over 70 are disposed to decline mammographic screening invitations but also if life expectancy is increasing.

Studies have shown it to be beneficial to screen women aged 40 to 49 [26, 54, 55] and to attend mammographic screening in general [56], but some have been critical with regard to the efficacy of screening women in that age group in comparison with other age groups [57], and also of the benefit of mammographic screening in general [58]. Questions have also been raised about the age limit, and there is support for screening women 75 years of age or older.

Nevertheless, it has been shown that screening is still important for early treatment [59], and the American Cancer Society recommends an annual mammography for women aged 40 or more, which should continue for so long as they are in good health [60]. The Swedish Cancer Society also addresses the question of raising the age limit as approximately 20 % of women diagnosed with breast cancer are 75 years of age or more [61]. This is noteworthy, since it may be a consequence of women in the oldest age group declining their invitation to mammographic screening, and is also an indication of the need for a higher upper age limit. As well as women in the oldest age group, this study identified another age group, 50–54 years, where distance appears to affect non-attendance. This may be of interest to investigate further, since some of the municipalities show a lower non-attendance rate than the reference municipality and for this age group. Equity in health concern everyone’s right to attain their full health potential and not be deprived this due to their social class or other social determinants [12], such as age and municipality of residence.

The socio-demographic characteristics for each municipality in this study provide a point of discussion in relation to previous studies. These characteristics, when influencing health, are often referred to as determinants of health (or the root causes). With this knowledge of previous findings and the determinants of health [12], certain of the socio-demographic characteristics for some of the municipalities in relation to non-attendance, fail to correspond. For instance Municipality B has the lowest rate of non-attendance (17.6 %), while having the highest proportion of foreign-born women in the female population in the age group 35 or older (25.83 %), the highest number of days of sick-leave, and also the lowest number of women with high education (10.92 %). However, previous findings, whereas being foreign-born [13, 25], being on sick-leave [33] and not having high education [13, 18] have been regarded as factors related to non-attendance of mammographic screening. The socio-demographic profile of this municipality (B) in comparison with the other municipalities, shows that, it has the highest proportion of women (83.17 %) who own a house or are shared owners in a cooperative building society. This may be interpreted to mean that women in this municipality are in a better socio-economic situation, which, according to previous research, has a positive effect on attending mammographic screening [18, 25, 30]. Interestingly is also that Municipality B is located >20 km from the screening facility. Another example is Municipality H, where the non-attendance rate is 19.6 %, but which is located at a distance among the furthest away from the mammographic facility. This municipality (H) not only has the next highest proportion of foreign-born women in the female population age group 35 or older (24.7 %), the next highest proportion of women with low education, and the highest proportion of unmarried/divorced/widowed (47.70 %), but also has the next highest proportion of women who own a house or are occupants in a cooperative building society. Furthermore, as shown in Table 4, Municipality H is the only municipality where non-attendance with regard to age and distance is significant for women in as many as four of the seven different age groups. The socio-demographic characteristics for these two municipalities offers a direction for further research in how they may exert influence on attendance and consequently be determinants of social equity in health.

### Strengths and limitations of the study

One strength of this study is that objective data from the mammographic clinic were used rather than self-reports. It also provides data on the total population of women eligible for screening in a Swedish county. The data used for socio-demographic variables were retrieved from Statistics Sweden, and are considered reliable, valid, impartial, and of high quality. Additionally, the data have already been collected, which facilitates a relatively quick overview of the situation. Their usefulness, however, are limited as the level of aggregation, at a certain level, provides a picture of the situation painted in very broad brush strokes in order to prevent any threat to individual privacy [62]. Another limitation of the study is its cross-sectional design, which does not allow for the associations of non-attendance with age and distance to be studied over time. The restricted number of individual variables for which data are accessible from the RIS is also a weakness. Further, the number of pairwise Chi-square tests conducted in Table 4 are not adjusted for multiple comparisons [63] as it seriously would increase the probability of Type II error.

In order to investigate the effects of socio-economic factors on non-attendance in greater depth we would need data at individual level.

## Conclusions

There is an association between municipality, and non-attendance of mammographic screening. Considering that there is one sole and pre-selected mammographic facility in the county, distance between the municipality of residence and the municipality where the mammographic facility is located may affect attendance rate.

There is also a positive effect on attendance of being older in comparison with younger (being in the youngest age group, 40–44). This, however, is not seen for women in the 70–74 age group.

Additionally, municipality of residence and greater age relate to each other regarding non-attendance, but not in all cases. From the perspective of equity, a lack of equal access to health and health care influences the utilization of health care facilities.
